# Xavier Bichat and the renovation of the pathological anatomy

**DOI:** 10.1177/09677720221097795

**Published:** 2022-05-02

**Authors:** Hélène Perdicoyianni-Paleologou

**Affiliations:** Department of Sciences and Technology Studies, 4919 UCL, London, UK

**Keywords:** Anatomy, pathology, physiology, tissue, vitality

## Abstract

Xavier Bichat, who lived a short life (1771–1802), was prominent French anatomist and physiologist during the time of revolution and one of the founders of French scientific medicine. He played a key role in the creation of the science of histology. Indeed, he was the first to see the organs of the body as being formed through the specialization of simple, functional units (tissues). Bichat is also known as one of the last of the major theorists of vitalism.


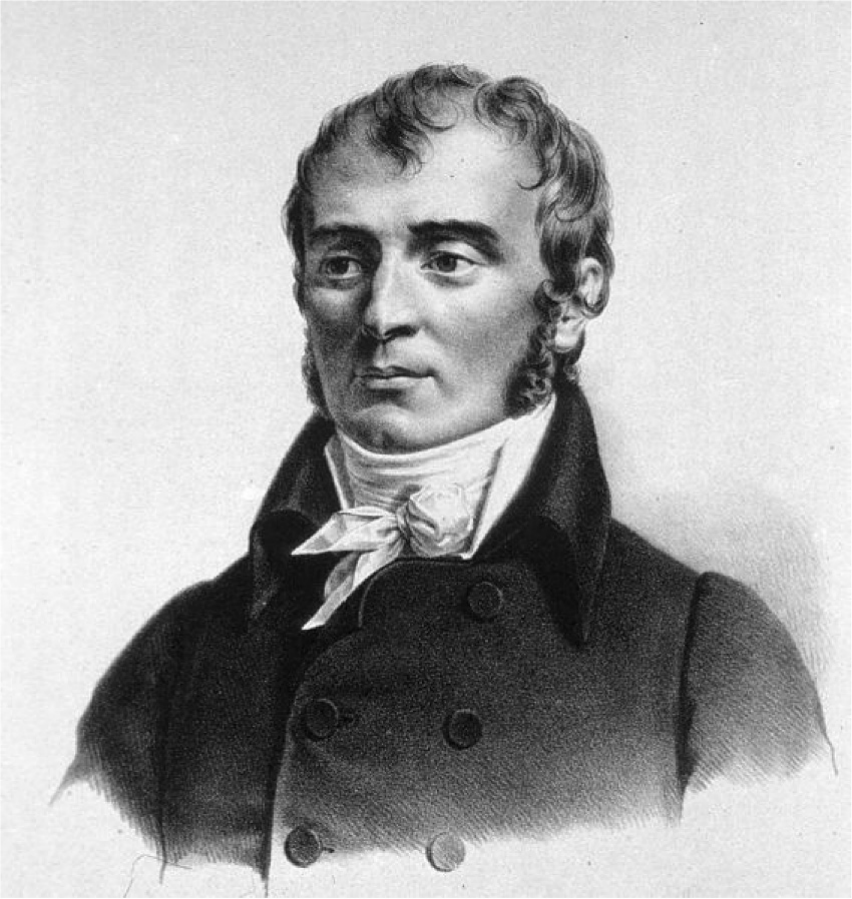


## Life and career

French anatomist and physiologist, Marie François Xavier Bichat was born in Thorette on the 14^th^ of November 1771. He spent his childhood with his family in Poncin. His father, Jean Baptiste Bichat, was his first teacher in anatomy. In 1782 Bichat entered the College of Nantua.

In 1790–1792 he studied philosophy^1^ and rhetoric^2^ at the *Séminaire de Saint Irénée* in Lyon as well as anatomy and surgery under the surgeons Marc-Antoine Petit (1766–1811) and Jean-Marie Viricel (1773–1855) at Hôtel-Dieu.^3,4^

In 1792 he worked as physician at the *Séminaire de Saint Irénée* and wrote many articles on diverse subjects, such as femoral fractures, polyps cure, humerus dislocation etc.

In 1793 he participated in the rebel movements, and shortly thereafter he returned to Poncin. In September of the same year, he was appointed to the *Hôpital de Bourg*.

Upon his arrival in Paris in 1794, Bichat was enrolled in Pierre-Joseph Desault's course at the ‘Grand Hospice de l’Humanité’ (later called ‘Hôtel-Dieu’)^5^ and served as an assistant to him.^6^ Desault was most impressed with the brilliant genius of his pupil and decided to take him into his house where he treated him as an adopted son.^2,6^ At the same time, Bichat pursued his research in anatomy and physiology.

Following the sudden death of Desault on June 1^st^ 1795, Bichat acquitted himself of the debts he owed to his benefactor by taking care of his widow and her son. He also completed the fourth volume of *Journal de Chirurgie*, that Desault founded, and gathered in one volume the surgical doctrines which his *Maître* had published in various periodical works.^6^

In 1796–1797 Bichat gave his first course of anatomical lectures.^6,7^ Being successful in this course, he decided to enlarge his circle of lectures, and to teach courses of operative surgery and physiology.^8^ But a sudden attack of hemoptysis forced him to interrupt his teaching for a short period. Once the danger had passed, he committed himself to working again as hardly as before.^1,6^

In 1799 Bichat lectured on the animal economy. Outlines of these lectures were published in the *Mémoires de la Société Médicale d’Émulation*, an institution established by him to promote professional standards in medicine, and afterwards elaborated in the *Traité des membranes en général et de diverses membranes en particulier*. During this year, he also published several articles on various topics in the same journal, such as the use of the trephine, polyp ligature, synovial membrane, membrane structure etc.

From 1799 onward Bichat abandoned surgery and did only research in anatomy, performing without using a microscope as many as 600 autopsies in a single year. This led him to understand the connection between disease and observable changes in the tissues. As a result, he considered useful to encourage doctors to autopsy the bodies of their patients to study the physiological effects that their illnesses had on the tissues of the body. He also determined with great precision the effects of remedial agents and instituted with this view a series of direct experiments which produced a vast store of valuable materials.

In 1800 Bichat he was appointed to serve as secretary of a medical advisory board established by the French government. Besides, he produced *Recherches physiologiques sur la vie et la mort*. This work was followed by *Traité d’anatomie descriptive* (1801) and *Anatomie générale appliquée à la physiologie et à la médecine* (1804). In 1825 F. Boisseau published Bichat's last course on *Anatomie pathologique*.

On 8^th^ July 1802, Bichat fell down the staircase at the Hôtel-Dieu. On the 15^th^, he became unconscious. Bichat died on the 22^nd^ of July 1802 at the age of 31.^1^ The cause of his fall remained unexplained. The day after his death, Jean-Nicolas Corvisart (1755–1821) wrote to Napoléon Bonaparte: ‘Bichat has just fall upon a field of battle which counts more than one victim; no one has done so much, or done it so well, in so short time’.^9^ Napoleon ordered a bust of Bichat and Desault to be placed in the Hôtel-Dieu. Under his bust is written ‘The Napoleon of Medicine’.^10^ Bichat's funeral was celebrated on the 23^rd^ July at the Notre-Dame. He was buried at the cemetery of Sainte Catherine. In 1803 his corpse was exhumed and transferred to the cemetery of Père Lachaise. A statue of Bichat by David d’Angers was erected in the courtyard at number 12 Rue de l’École de Médecine in Paris and another stands at 15–21 of the same street. This statue has undoubtedly served as a model for the one placed in Bourg-en-Bresse.

### General anatomy *versus* descriptive anatomy *versus* physiological anatomy

According to Bichat, general anatomy is the branch of medicine concerned with the study of the simple tissues, which are structural and vital elements. Tissues are simpler than organs and consist of combinations of interlaced vessels and fibers. The human body is resolved into 21 different kinds of tissues, including nervous, vascular, and mucous tissues.

Descriptive anatomy is the part of anatomy which treats of the forms and combinations of simple tissues. Organs are made up of assemblages of tissues and are components of more complex entities called ‘organ systems.’ The combination of several organs being charged with the performance of a special function is named *appareils* (*apparatus*).

Bichat therefore goes from the isolated history of each of the major elements that form part of the structure of the *appareils* to the description of these *appareils* and to that of the functions and properties of the organs that constitute them. General anatomy is, in this respect, an essential introduction to descriptive anatomy and physiological anatomy.^11^

### General anatomy: tissular theory and method

Although he was strongly influenced by Étienne Bonnot de Condillac's sensualism (1714–1780) and his analytical method, which was later adapted to medicine by Philippe Pinel (1745–1826), Bichat rejected their nosological speculations. However, he kept Pinel's theory of mucous membrane lesions, which he developed further by defining general anatomical categories as kinds of tissues. To this end, Bichat used criteria of spatial continuity.^11^ He localized the tissues inside cavities in continuity with the skin first and then divided them into two groups, whose tissues were in continuity. Thus, a first group is in the interior the nose, mouth, pharynx, larynx, esophagus, stomach, intestine, and anus. A second group is in the urethra, ureter, prostate and vagina.

Bichat's anatomical studies gave birth to *General Anatomy*, which is considered one of the foundations of today's medicine. In this work, Bichat investigates the following points:

- the forms of the cellular system, its organization, its properties, its development; the tissues of the cellular system outside each organ, those of the cellular system inside each organ; tissues of the cellular system considered independently of the organs;
- the external forms of the nervous system of animal life, its organization, its properties, and its development;
- the ganglia and nerves that are part of the nervous system of organic life;
- the forms and general structure of the vascular system with red blood and that with black blood, their organization, their properties, and their development;
- the structure of the general capillary system and that of the pulmonary capillary system, as well as their properties;
- the structure of the exhaling system, its properties, its functions and its development;
- the origin of absorbent vessels and lymphatic glands, their forms, their structures and their properties.

Due to his untimely death, Bichat did not have the opportunity to do a fine dissection to thoroughly study the anatomical tissues. His observations are mainly based on experimentation. Thanks to his clinical observations, he defined, in terms of normal anatomy, the tissue as the last element of the anatomical analysis of bodies. In respect of pathological anatomy, he showed that diseases are a modification of life itself and result from the lesions of various tissues.^12,13^ He therefore subdivided carditis into pericarditis, myocarditis, endocarditis; brain inflammation into meningitis, encephalitis, arachroiditis; eye diseases into conjunctivitis and retinitis; pathological changes in cellular tissues into fatty degeneration, development of scar tissue, senile sclerosis and cancerous tumor.

According to Bichat, the origin of skin scars is directly related to the structure of cellular tissue. Within the process of repairing skin wounds, he distinguished between healing by secondary intention (*sanatio per secundam intentionem*) and healing by primary intention (*sanatio per primam intentionem*). *Sanatio per secundam intentionem* is divided into four periods: inflammation, appearence of pimples, suppuration, elimination of pimples and appearance of the scar membrane. During *sanatio per primam intentionem*, the three last periods disappear. The stage of inflammation is followed by that of tissue healing. Cellular tissue influences on scar formation as well as on tumor and cyst formation. This highlights the cellular nature of all tumors and cysts.

Bichat classified disorders according to an anatomy which is different from all treatises of his time. By examining both healthy and sick anatomical organs and tissues, he defined general and real anatomical categories, which were undefinable with anatomy, physiology, or pathology alone.^14^

### Descriptive anatomy: anatomic division and method

The anatomical division that Bichat uses in his work *Descriptive Anatomy* to describe the various combinations of simple tissues is based on the criterion of classification of the functions of the *appareils*, of which he distinguishes three categories:

- the *appareils* of the animal life, that provide the ability to interact with other beings;
- the appareils of the organic life, that serve for the constant composition and decomposition of the body;
- the reproductive *appareils*.

The *appareils* of the animal life are as follows: the locomotor *appareil*, which permits movement from a place to another. The key components of this *appareil* are the bones and the muscles; the vocal *appareil* (larynx); the external sensitive *appareil* (the eyes, the ears, the nostril, the tongue and the skin); the internal sensitive *appareil* (the brain and its membranes, the spinal cord and its membranes); the emotion and motion transmission *appareils* (the brain nerves and the ganglion nerfs).

The *appareils* of the organic life are the following: the digestive *appareil* (the gastrointestinal tract and the accessory organs of digestion); the respiratory *appareil* (the trachea, the lung and its membrane); the circulatory *appareil* (the heart and its membrane, arteries, the veins of general system and those of abdominal system); the absorbent system (absorbents and glands of absorbents); the secretory *appareil* (tear ducts, salivary and pancreatic ducts, biliary ducts and urinary ducts).

The reproductive *appareils* are subdivided into two categories: the male reproductive system, which includes the penis, testicles and its membranes, and the female reproductive system, which is made of external organs and vagina, and of the uterus.

As we have indicated, Bichat was a pupil of Desault, whose teaching on anatomy was purely descriptive and marked by extreme precision. Desault described the form, position and function of each organ by locating it on the seven planes of space. This descriptive approach was finalized by the definition of the concepts of functional mechanics, pathological anatomy, and surgical anatomy. Desault's exhaustive description was not imitated by Bichat, who provided little detailed descriptions that aim at emphasizing the essential points, avoiding excessive precision.

The originality of Bichat's theory lies in the following:

- He rejected the theory of his predecessors maintaining the union of organs;
- Considering descriptive anatomy as the first step in the study of organ functions, he postulated that the functions themselves should serve as a means of dividing the *appareils* that perform them;
- While opposing the general outline of descriptions, he put forward that each *appareil* must have a different descriptive mode;
- He was strongly opposing the detailed descriptions of his predecessors and contemporaries, because ‘elles tuent le génie sans soulager la mémoire;^15^
- He laid the foundation of surgical anatomy by placing great emphasis on the position and relation between organs;
- He combined human anatomy adult with ‘l’anatomie comparée des divers âges’;^16^
- By observing the movements of animals, he described the functions of bones and muscles. In doing so, he contributed to the development of functional anatomy, introduced by Jean-Bénigne Winslow (1669–1760);
- He invented a nomenclature based on the position of the organs. In addition, he favored the creation of an anatomical language expressing many objects by a small number of terms;
- He took a stand against the following educational tools: a) plates and ceratoplastic preparations, which are ‘inutiles à celui qui sait, et souvent nuisibles à celui qui ne sait pas’^17^ and ii) the vast knowledge which is acquired by reading books and belongs more to the history of science than to science itself. By maintaining that observation is the basis of most physical sciences and that only bodies tell the truth, Bichat is regarded as the founder of practical medicine, which represents a real revolution in medical studies.

Through the description based on the observation of the external form of organs, their position and their structure, Bichat reveals himself to be the founder of functional anatomy. His descriptive method is marked by conciseness and accuracy.

### Vitality, life and death

The *Recherches physiologiques sur la vie et la mort* [*RP*], which was first published in July of 1801, is divided into two parts. The first part presents Bichat's philosophical researches on life and the second one, his experimental researches on death.

In the philosophical researches on life Bichat divided the organs of the body into external ones which interrupt their activity for a certain amount of time and internal ones which function continuously. In addition, he made the distinction between animal life, aka external life, and organic life, alias internal life.

## Animal life *versus* organic life

The animal life encompasses the central nervous system, the brain and the spinal cord. It is the order of active and reactive interaction with surrounding things and conditions. That is, when the animal life is present in a body, the animal establishes relationships between itself and the environment. It becomes conscious; it has emotional reactions guided by sensory information; it moves voluntary and conveys its feelings. The animal life is symmetrical, i.e. it possesses regularity of form of which the brain is the height. It operates harmoniously and at irregular intervals; it begins at birth; it possesses the capacity to be educated and to expand its natural limits.

Throughout the *RP*, Bichat strove to naturalize the history of human progress in accordance with the function of animality. By setting the animal life in the cultural mode of citizenship (‘être l’habitant du monde’^18^^)^ and that of conjugality (‘marier son existence à celle de tous les autres’,^19^^)^ Bichat ‘socializes animality as a physiological class.’^20^ Besides, he believes that the nature that modulates the growing complexity of animal life and, at the same time, operates on the already cultural modes of relation with social environment is on its own well-ordered and rational. In Bichat's theory, culture has an effect on the perfection of the organs. This explains how some people achieve ‘une perfection qui ne leur est naturelle et qui les distingue spécialement des autres.’^21^ Thus, education and culture complete and bring to perfection the specialization natural to animal life.

The organic life is passive and, to a great extent, unobserved. It is the internal order that is involved in both vegetal and animal life forms. The organic life is focused on the heart. It is asymmetric and does not necessitate regularity of motion. It is restricted to the interior, to the entirely nutritive. It is present from inception of pregnancy and cannot expand its natural limits. Thus, it is not subject to any mode of cultural and social influence. However, it is susceptible to the influence of passion-inspiring objects. Passion is a uncontrollable intruder which invades the body through the humors and leaves an everlasting impact on the organic functions with the appearance of temperament and character.

## Sensitivity *versus* contractility/irritability

Bichat rejected the explanation provided by many physicians of Montpellier that vital phenomena are the result of soul or larger vital forces. He posited that vital laws, such as sensitivity and contractility/irritability, need replace souls and abstract vital forces. These vital laws can be experimentally observed and thoroughly investigated.

In examining the function of the body, Bichat realized that the forces of sensitivity and contractility/irritability are present within organic and animal life.

In organic life, sensitivity is ‘the faculty of receiving an impression’, and in animal life, ‘the faculty of receiving an impression and moreover of referring it to one common center.’^22^

In organic life, the operation of sensitivity and the stimulation of the contractility/irritability induced by it derive from the ‘*rapport* which exists between the sensitivity of each organ, and bodies which are external to it.’^23^ As a result, organic sensitivities cause immediate contractilities/irritabilities in the tissues and the organs with which they are connected.

By contrast, animal sensitivities send impressions to the brain. The brain is conscious of these sensitivities, but it may or may not pursue them to enkindle animal contractilities/irritabilities. The sensitivities do not in themselves produce any action.

In Bichat's system, sensitivity in any organ is capable to activate efficiently a corresponding contractility. Organic sensitivity is divided into ‘sensible organic contractility’ and ‘insensible organic contractility’:

Sensible organic contractility is observed in the heart, stomach, intestines, bladder, etc. and exercises itself on the considerable masses of animal fluids.

Insensible organic contractibility is that by virtue of which the excretory ducts react to their fluids, the secretory organs to the blood which comes to them, the parts in which nutrition operates on the nutritive juices, and the lymphatics upon the substances which excite their open extremities, etc. Whenever fluids are disseminated in small masses, or where they are minutely divided, there this second kind of contractility is displayed.^24^

Since the Seventeenth Century, the forces of sensitivity and contractility/irritability have been considered as the basis of life. This theory has been developed by Albert von Haller (1707–1777), Francis Glisson (1597–1677), Théophile Bordeu (1722–1776) and Robert Whytt (1714–1764).

In several places, Bichat admitted that he was much influenced by these scientists. However, this did not prevent him from revising and declining many principles of their theories.

Like Glisson, Bordeu and Whytt, Bichat considered sensitivity as a necessary condition for contractility/irritability in all kinds of structures.

Unlike Haller and Whytt, who maintained that sensitivity is a merely conscious feeling, Bichat believed that sensitivity is unconscious, namely, organic.

In contrast to Haller, who ruled in favor of a rigid categorization of organic reaction, Bichat pointed out that the animal and organic contractilities/irritabilities are closely related between them. Like Whytt, Bichat stated that every organ is subject to sensitivity. This explains how inflammation of any organ causes organic sensitivity and thereby animal sensitivity.

Bordeu's influence on Bichat is evident through the latter's theory on organic sensitivity, especially the affinities or sensitivities of certain organs for particular substances. By having recourse to a quality almost identical to that which Bordeu has attributed to glands, Bichat explained the way how intestinal lacteals soak up only chyle and the larynx permits only air to enter it. The alteration of these affinities raises inflammation. Inflammation results from the dysfunction of laws determining the passage of fluids into canals.

In conclusion, Bichat founded a new science of the nature of the living organisms. His theory on vitalism was based on the concept that vital doctrines overrule physical laws.

## Life *versus* death

In the Preface to the *RP*, Bichat defined life as the collection of those functions which resist death. Life is a reaction of living body against the forces of decomposition which attacks it and finally triumphs over it. Life and death are two distinct categories, each having its own force. The organism is an island of vitality as long as vital forces persist

In the experimental researches on death, Bichat illustrated the independent role of the heart, the lungs, and the brain in the functioning of the physiological systems. The heart supports life by pumping blood to the tissues of the body. The brain takes action in the nerves, by the help of galvanism. The lungs supply the body with nourishment and avert poisoning by their action on the blood.

The first part of the *RP* deals with the effect of the death of the heart on the brain, the lungs and the orther organs.

As a first step, Bichat observed the physiological dependency of the brain on the heart:

Now the heart can only act upon the brain in two ways: namely, by the nerves, or by the [blood] vessels which serve to unite them. These two organs indeed have no other means of communication.^25^

The blood vessels are another means of communication between the heart and the brain:

The ventricle and auricle [pumping] red blood manifestly influence the brain by the fluid carried there by the carotid and vertebral arteries. Now this fluid, on arriving there, may excite the brain in two ways: first, by the motion with which it is agitated, and second, by the nature of the principles, which constitute it, and which distinguish it from the [venous] black blood.^26^

As a result, ‘the motion of the blood, by communicating itself to the brain, keeps up the action and sustains the life [of the brain]’.^27^

In short, Bichat discovered the primary and fundamental function of the heart, which is to sustain the tissues of the brain by agitating them. Any interruption of the normal supply of the blood to the tissues of the brain breaks off the activity of the brain at once.

The understanding of the physiological relations between the heart and the brain led Bichat to determine the anatomy of the cranium and the blood vessels conducting blood to it. The outer, convex shell of the skull directs arterial motion toward the tissues of the brain. The veins, carrying a continuous stream of blood gradually away from the brain, are located against the inner, concave surface of the skull.

As a second step, Bichat examined the effect of the heart on the lungs, which is not as direct as that of the heart on the lungs.

Bichat first explained the function of the lungs:

The lungs are the seat of two very different species of phenomena. The first, which is entirely mechanical, relate to the motions of elevation or depression of the side of the diaphragm, to the dilation and collapsation [*sic*] of the air vesicles, and to the entrance and egress of the air, the effect of these motions. The second, purely chemical [phenomena], relate to the different alterations which the air experiences, and to the changes in the composition of the blood, etc.^28^

Second, he demonstrated how the heart pumps ‘black’ blood straight to the lungs, enabling them to use their chemical function, which is ‘to imbibe new principles from the air, and to communicate to it those with which [the blood] is surcharged.’^28^ Should the stream of the blood from the heart be interrupted, the lungs cease to use their chemical function. The cessation of the mechanical function of the lungs therefore follows upon the death of the heart which eliminates the substance on which they act.

As a third step, Bichat explored the effect of the death of the heart ‘upon all the organs’ and ‘upon the general death’. The interruption of the circulation of blood resulting from the death of the heart in the organs that are supplied with it is the cause of their failure and death in a brief amount of time.

The second part of the *RP* explores the effects of the deaths of the lungs and the brain.

Having observed the stream of the blood through the lungs after the death of the diaphragm and the distention of the heart when the lungs normally function after the death of the heart, Bichat concluded that ‘the interruption of the mechanical phenomena of respiration does not directly produce a cessation of the action of the heart.’^29^

Bichat then investigated the death of the lungs. This is caused by the distribution of the black blood to the pulmonary tissues, which is resulting from the interruption of the chemical functions of the lungs. The infusion of the black blood to the tissues of the heart, the brain and the other organs breaks of their function as well. His statement is formulated as follows:

… in the interruption of the chemical phenomena of the lungs there is a general effect on all the parts, that the black blood, driven everywhere, carries weakness and death to every organ that it enters; that is not from their not receiving blood, but from their not receiving red [blood], that each organ ceases to act; and that, in a word, all [tissues] then find themselves penetrated by the material cause of their death, namely, black blood.^30^

In addition, Bichat described the effect of the death of the brain on the lungs:

The moment the brain ceases to act, the lungs suddenly interrupt all their functions. This phenomenon, which is universally observed in animals with red and warm blood, can happen only in two ways: first, because the action of the brain is directly necessary to that of the lungs: or second, because the latter receive an indirect influence from the former through the intercostal muscles and diaphragm, which influence ceases when the cerebral mass is inactive. Let us inquire which of these two modes is that fixed on by nature.^31^

For Bichat, according to the laws of nature, the great sympathetic nerve ‘is only an agent of communication between the organ and the ganglia, and not between the brain and the organ.’^32^ Besides, any inflammatory disorder or section of the vagus nerve cannot cause a death blow to the lungs. The death of the brain does not therefore cease the function of the lungs immediately. In fact, the mechanical action of the lungs is interrupted by the influence exerted by the death of the brain through the mechanical motion of the intercostal muscles and diaphragm. Bichat clearly demonstrated how the motion of the muscles is ceased when the nerves running between them are sectioned.

In sum, the investigation of the three vital systems, i.e. the heart, the brain and the lungs, led Bichat to hierarchize them following the limits and the scale of their actions and effects. For the first time in the history of medicine, there is a ‘conceptual mastery of death’^33^ and an unveiling of its gradual process: not all the organs die at once.

In conclusion, Bichat is regarded as a foremost figure of medicine in the creation of a new concept of anatomy. He formed the concept of generality, which is manifest in the definition of general anatomical categories as kinds of tissues. Moreover, he largely contributed to functional anatomy by providing an accurate and comprehensive description of the structure and functions of the organs.

Besides, Bichat established physiology as a science irreducible to the physical sciences. The aim of this concept was to explain the all-important properties of animality in the light of the following antithetical processes: animal/external life, sensitivity *versus* organic/interior life, contractility; life *versus* death.
